# Stimulus statistical context sensitivity of deviant responses to auditory intensity changes

**DOI:** 10.1038/s41598-026-55029-3

**Published:** 2026-06-04

**Authors:** Maryam Aghamolaei, Abishek Umashankar, Ekaterina Yukhnovich, Maria Chait, Timothy D. Griffiths, Kai Alter, William Sedley

**Affiliations:** 1 Faculty of Medical Sciences, Newcastle University, Newcastle upon Tyne, UK; 2 University College London, EAR Institute, London, UK

**Keywords:** Mismatch negativity, Deviant, Prediction error, Predictive processing, Loudness, Intensity, Evoked potentials, Neuroscience, Psychology, Psychology

## Abstract

Mismatch negativity (MMN) is widely used to study sensory function and its clinically-relevant perturbations, and is modulated by the preceding statistical stimulus context, e.g. standard deviation (SD) of tone frequencies. It is unknown whether similar statistical modulation applies to changes in stimulus intensity; whilst MMN is elicited by intensity changes, intensity is encoded differently to other sensory features, including non-topographic organisation, and absence of single-unit responses to intensity decreases. In two related EEG experiments on normal-hearing volunteers (*n* = 17/15), we replicate findings from other stimulus properties, showing that MMN to intensity deviants is larger within a low-SD stimulus context. However, we found several differences between high- and low- (vs. standard) intensity deviants. Statistical modulation of high-intensity MMN began earlier, and was robust across both experiments. Low-intensity MMN modulation began later, and was only elicited by one of the two experimental paradigms, which we suspect indicates that adaptation over long timescales and/or higher cortical levels may be required for its statistical modulation.

## Introduction

Detection of regularity and generation of predictive models are thought to be basic functions of sensory systems^1,2^. As such, the brain is exquisitely sensitive to deviations of sensory input from established patterns or prior predictions. A commonly used marker of such ‘deviance’ is a negative scalp potential occurring from around 150 ms termed ‘mismatch negativity’ (MMN)^3,4^. Whilst elicited by various types of deviance in different sensory domains, MMN has been most studied in the auditory system in response to changes in stimulus frequency. The classical paradigm used to elicit MMN is the ‘oddball’ paradigm (a series of identical repeating ‘standard’ stimuli interspersed with less frequent pseudo-random instances of ‘deviant’ stimuli), but numerous variations and alternatives have been implemented^4^. Typically, MMN is calculated by subtracting from the deviant response the response to the physically identical standard stimulus. Source modelling of auditory MMN has predominantly implicated a bilateral network comprising primary and non-primary auditory cortex and inferior frontal gyrus, though other brain areas likely contribute^5^.

Conditions resulting in MMN also elicit transient firing rate increases in individual neurons throughout the central auditory hierarchy^6^. By comparing different experimental contrasts, these have been separated into responses representing stimulus-specific adaptation (SSA), driven by deviant stimuli simply being less common and therefore less adapted to than standards, and ‘prediction error’ responses, reflecting the prior improbability (or ‘surprise’) associated with the deviant stimulus. In general, higher vs. lower hierarchical level, and extralemniscal vs. lemniscal location, are associated with a greater proportion of MMN being accounted for by prediction error. Whilst such single-neuron deviance-associated responses are elicited by changes in various auditory properties (e.g. frequency, location, duration), they have only been observed in response to increases in intensity, but not decreases. Furthermore, in the inferior colliculus, stimulus-specific adaptation has been found to occur to acoustic stimulus frequency but not to intensity^7^. Conversely, scalp-level MMN is reliably observed to decreases in stimulus intensity^8^, as well as increases^9^. Studies have variably reported that MMN elicited by increases and decreases differ in their morphology^10^, for instance with only increases eliciting a larger N1 or a P3a response^11^, or being similar in latency and amplitude^12^. Paradigms used in MMN studies such as these often differ, including some where intensity is the only varied stimulus features and others where multiple attributes are varied. We have previously observed that even the presence of a different stimulus frequency in another part of the experiment can affect the relative magnitude of intensity increase vs. decrease MMN responses^13–15^.

It has been found that MMN responses to stimulus frequency changes, recorded with magnetoencephalography (MEG), are sensitive to the statistical properties of the population of standard stimuli in which they are embedded^16^. This approach compared identical standards and deviants, but occurring within two different Gaussian distributions from which all other stimuli were drawn; these had the same mean but differed in standard deviation. The small number of standard stimuli were placed at the exact mean of the Gaussian distributions, such that identical standard and deviant stimuli could be compared across the two Gaussian conditions. Larger MMN responses were observed in the low standard deviation condition, which, mechanistically, could indicate greater statistical surprise and/or reduced adaptation to the deviant frequency. This paradigm has since been used in further studies, including with electroencephalography (EEG)^17^.

In this study, we focussed on using the same approach to study MMN responses to intensity deviants (intensity MMN), with two principal questions in mind:

- Is intensity MMN modulated by the statistical context of the stimuli in which deviants are embedded (i.e. the standard deviation of the intensity of preceding stimuli)? This is not a trivial question, because SSA should vary relatively little between statistical contexts, due to all stimuli being the same frequency^7^. Furthermore, the extent to which predictive processing of intensity mirrors that of frequency has not been extensively studied.
- Are there asymmetries in MMN responses to upward compared to downward changes in intensity, and how these are modulated by statistical context? Again, this is non-trivial, as single neurons responsive to intensity decreases have not been demonstrated in the auditory pathway^6^, and because intensity decreases do not necessarily involve the activation of any additional neurons (unlike intensity increases, which lead to activation of more off-frequency neurons), or any increase in the strength of input to any auditory neurons already recently activated.

## Methods

### Participants

Participants were healthy young adults, with no known hearing difficulty, recruited via local advertisement. Two versions of the experiment were run, with 17 participants completing Experiment 1 and 15 completing Experiment 2, of whom 8 completed both versions (on separate occasions). All participants provided informed consent, and the study was conducted in accordance with procedures given ethical approval by Newcastle University (ref 10356/2018), and in accordance with the Declaration of Helsinki.

### Experimental paradigm

Experiments took place in a dimly-lit soundproof room, and included pure-tone audiometry before the main experiment, in octave steps from 0.25 to 8 kHz. The main experiment was conducted under passive listening conditions, whilst participants watched a silent subtitled television show or movie of their choosing.

**Fig. 1:**
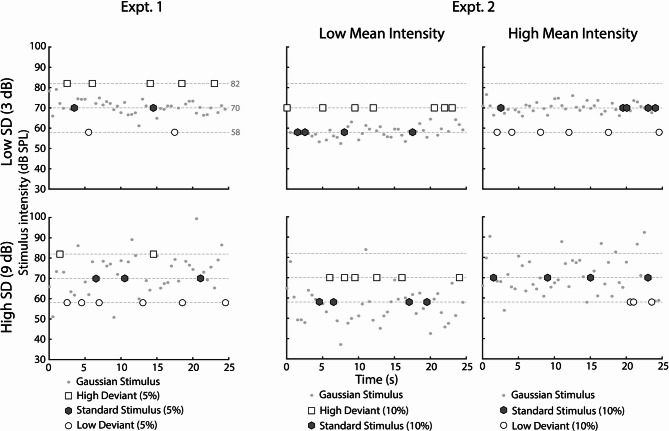
Paradigm for the two experiments. For each experiment and block type, the first 50 stimuli from one example subject are shown.

The paradigm (Fig. 1) was adapted from Garrido et al.^16^, with the principal change being that in our experiment stimulus frequency was fixed, and intensity varied as the experimental parameter. Stimuli were isochronous 1 kHz pure tones, delivered diotically through Sennheiser HD Pro over-ear headphones, of 50 ms duration, 10 ms onset/offset raised cosine ramps and 500 ms stimulus onset asynchrony (i.e. 450 ms inter-stimulus interval). Stimuli in each block of the experiment consisted of 85% Gaussian Stimuli (GS), 5% Standard Stimuli (SS), 5% Low Deviants (LD) and 5% High Deviants (HD), determined pseudo-randomly, ensuring these proportions were maintained exactly. No constraints were placed about which stimulus types could follow each other.

In Experiment 1, there were two block conditions: Low Standard Deviation (LSD) and High Standard Deviation (HSD), corresponding to 3 and 9 dB, respectively. GS were drawn from a Gaussian distribution with this SD value and a mean of 70 dB HL. SS were exactly 70 dB SL, LD were 58 dB HL, and HD 82 dB HL. There were 1,560 stimuli per block, and 2 blocks for each of the LSD and HSD conditions, with block order randomised. This resulted in 156 of each of SS, LD and HD for each of the LSD and HSD conditions.

Experiment 2 was identical to Experiment 1, except that as well as experimental blocks differing in SD, they also differed in mean stimulus intensity, leading to four combinations of SD and mean. There was one block for each of these combinations, and the proportion of standards and each type of deviant was increased to 10% to result in the same total number of each type of stimulus per condition as in Experiment 1, despite the halved number of blocks. Low and high SD remained at 3 and 9 dB, respectively. One further difference was that no HD stimuli were included in High Mean intensity (HM) blocks, and no LD stimuli in Low Mean intensity (LM) blocks. As evident in methods described later, these deviant stimuli were not required for any analysis, and were excluded to allow the number of necessary deviant stimuli to be maintained. HM blocks were otherwise identical to the corresponding blocks in Experiment 1. In LM blocks, the intensity of all stimuli was simply reduced by 12 dB, resulting in 58 dB for SS and the mean of GS, 46 dB for LD, and 70 dB for HD. This arrangement meant that LD stimuli in HM blocks were physically identical to SS in LM blocks, and that HD stimuli in LM blocks were physically identical to SS in HM blocks.

The respective advantages and disadvantages of the two experiment types are that Experiment 1 compares standards and deviants from relatively more equivalent states of auditory adaptation but that are not physically identical, whilst Experiment 2 compares physically identical standards and deviants but in different states of auditory adaptation. Featuring both experiment types aimed to help determine the extent to which differences in physical stimulus properties or state of adaptation could account for any results observed.

### EEG acquisition and preprocessing

EEG data were acquired with a 64 channel Biosemi ActiveTwo system at a sampling rate of 256 Hz, ensuring electrode offsets were maintained within the recommended range of +/-10 mV. Data preprocessing was conducted in Matlab using the EEGlab toolbox, comprising referencing to P9/P10 electrodes (similar to linked mastoids), (non-phase distorting) bandpass filtering between 1 and 35 Hz, independent component analysis (ICA) using the Infomax algorithm, removal of components containing predominantly ocular or cardiac artefacts (range 1–3 components per subject), interpolation of noisy channels, exclusion of bad time periods using the ‘clean_rawdata’ function on default settings, epoching to -100-500 ms peristimulus time, and baseline correcting to -100-0 ms. Data were restricted to the FCz channel for statistical analysis, and remained referenced to P9/P10.

### Statistical analysis

The principal statistical contrasts were the ones that reflected the difference in MMN magnitude between LSD and HSD blocks, as follows:

In Experiment 1: for upward intensity deviants, (HD_LSD – SS_LSD) – (HD_HSD – SS_HSD); for downward intensity deviants, (LD_LSD – SS_LSD) – (LD_HSD – SS_HSD); to expose differential effects dependent on deviant direction, ((HD_LSD – SS_LSD) – (HD_HSD – SS_HSD)) – ((LD_LSD – SS_LSD) – (LD_HSD – SS_HSD)), which simplifies to (HD_LSD – LD_LSD) – (LD_HSD – HD_HSD).

In Experiment 2: for upward intensity deviants, (HD_LSD_LM – SS_LSD_HM) - (HD_HSD_LM – SS_HSD_HM); for downward intensity deviants, (LD_LSD_HM – SS_LSD_LM) - (LD_HSD_HM – SS_HSD_LM); to expose differential effects dependent on deviant direction, ((HD_LSD_LM – SS_LSD_HM) - (HD_HSD_LM – SS_HSD_HM)) – ((LD_LSD_HM – SS_LSD_LM) - (LD_HSD_HM – SS_HSD_LM)).

Significant differences between conditions were determined using a Monte Carlo non-parametric permutation approach^18^ implemented in custom-written code. In each of 10,000 permutations per analysis, the stimulus type labels (encompassing stimulus type, block SD and block mean) were randomly shuffled. Data were then averaged across trials within each stimulus type label, and analysis otherwise proceeded as for the unshuffled data, as follows.

A one-tailed test was performed, considering only negative values of the contrast performed, in the time window 80 to 300 ms peristimulus time. This window was chosen to capture both classical MMN and earlier MMN in the N1 timeframe, whilst preserving statistical power by constraining the data space examined. The largest negative value in that timeframe for each permutation was added to the null distribution. Thus, all time points where the unshuffled averaged data had a value more negative than the 500th -largest negative value in the null distribution (i.e. *p* < 0.05) were considered to show significant differences.

We also performed some more basic contrasts to provide a more comprehensive analysis of the data, but which were not intended to address the research question. As such, we did not perform multiple comparisons correction other than the correction for multiple time points inherent in the Monte Carlo approach.

For Experiment 1, these secondary contrasts included: SS_LSD – SS_HSD (SD-related differences in standard stimuli); LD_LSD – SS_LSD (Low deviant MMN in low SD condition); HD_LSD – SS_LSD (High deviant MMN in low SD condition); LD_HSD – SS_HSD (Low deviant MMN in high SD condition); HD_HSD – SS_HDS (High deviant MMN in high SD condition). In the case of SS_LSD – SS_HSD, we were also potentially interested in positive differences, and therefore used two-tailed statistics (i.e. adding the largest absolute value in a permutation to the null distribution).

For Experiment 2, secondary contrasts included: SS_LSD_HM – SS_HSD_HM (SD-related differences in high mean intensity standard stimuli); SS_LSD_LM – SS_HSD_LM (SD-related differences in low mean intensity standard stimuli); LD_LSD_HM – SS_LSD_LM (Low deviant MMN in low SD condition); LD_HSD_HM – SS_HSD_LM (Low deviant MMN in high SD condition); HD_LSD_LM – SS_LSD_HM (High deviant MMN in low SD condition); HD_HSD_LM – SS_HSD_HM (High deviant MMN in high SD condition).

## Results

### Subject factors

All subjects had normal hearing thresholds, defined as < 20 dB HL at all frequencies between 0,25 and 8 kHz in both ears.

### Standard stimuli

**Fig. 2:**
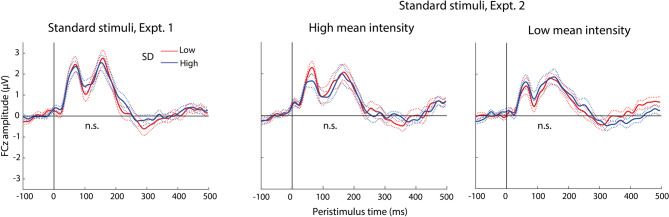
Responses to standard stimuli. Dashed lines indicate standard error. No significant differences between the two SD conditions were seen in either experiment-intensity combination. Prominent N1 responses were not observed, typical for studies using isochronous stimuli of unchanging frequency.

There were no differences between the amplitudes of responses to standard stimuli in the two SD conditions for Experiment 1, or between the amplitudes of responses to standard stimuli in the two SD conditions for high mean intensity, or between those in the two SD conditions for low mean intensity (Fig. 2).

### Mismatch negativity (MMN) responses within SD conditions

In Experiment 1, where standard and deviant stimuli were of different intensity, and for both SD conditions, P50 responses were of significantly smaller amplitude in low deviant than standard stimuli (74–78 ms for high SD, 74–82 ms for low SD). Note that much of this timeframe was outside the a-priori window of statistical interest (80–300 ms), so we interpret these results with caution. No differences in P50 responses were observed in Experiment 2, where standard and deviant stimuli were physically identical.

**Fig. 3:**
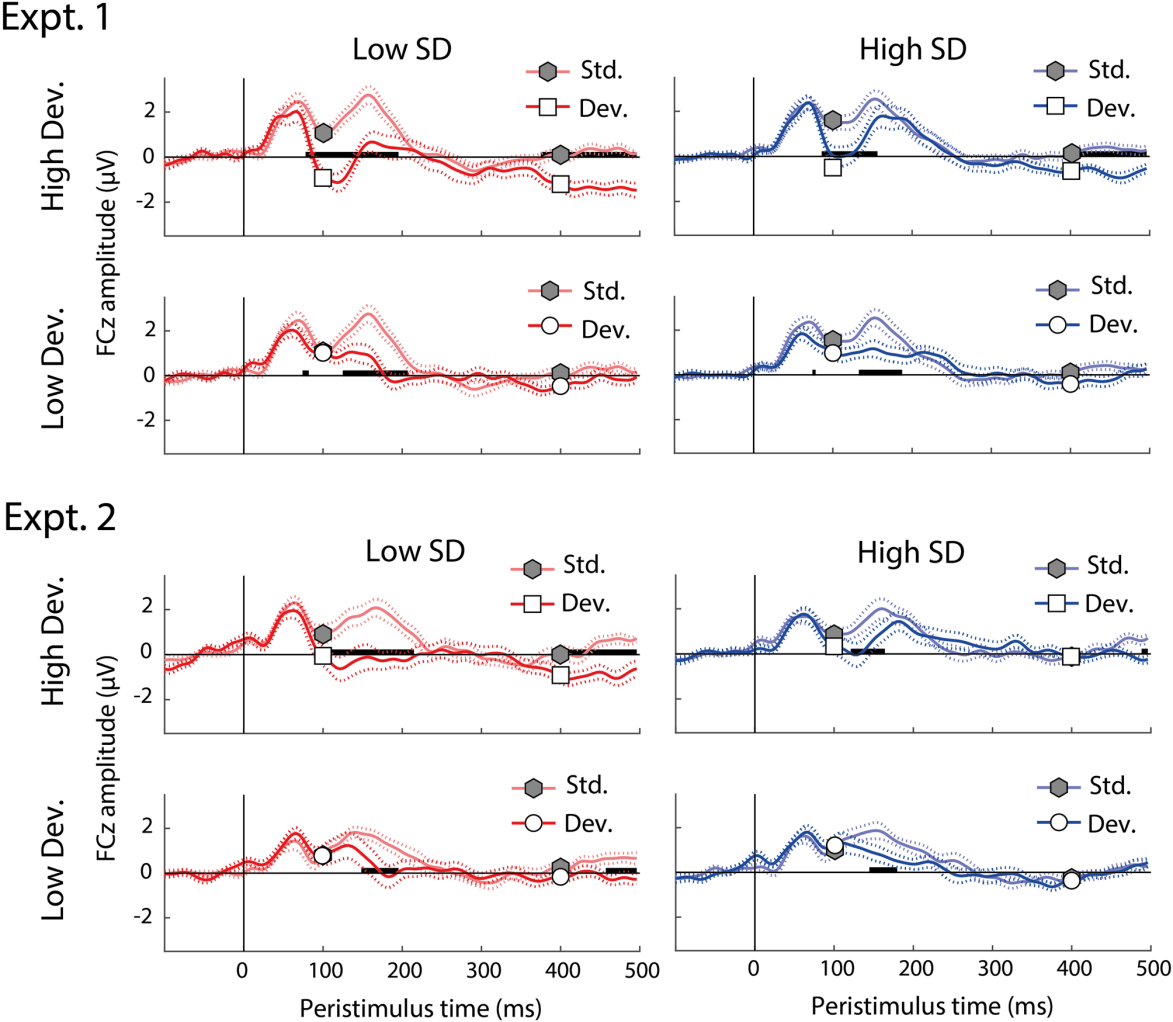
Comparison of standard and deviant stimuli. Dashed lines indicate standard error. Black bars indicate time points with significant difference based on non-parametric permutation analysis. In Expt. 2, standards were physically identical to corresponding deviants, on account of being taken from experiment blocks with the same SD but different mean intensity. Note: difference waveforms (Dev. – Std.) for these comparisons are displayed in Fig. 4.

Significant MMN was observed in both experiments, for both SD conditions and both intensity deviant directions (Fig. 3). In all instances, significant MMN began earlier for high deviants than equivalent low deviants. Particularly, but not quite exclusively, for high intensity deviants, and more so in Experiment 1, significant negativity was also observed in a second timeframe from around, or just before, 400 ms. A smaller but still significant negativity in the same timeframe was also observed for low deviants in the low SD condition of Experiment 2. Latencies in which deviant potential was significantly more negative than standard are summarised in Table 1. We note that these changes were outside the time window used for generating the null distribution, and exercise appropriate caution accordingly in the Discussion section.

**Table 1:** Latencies (ms) containing significant MMN. i.e. where deviant amplitude was significantly more negative than corresponding standard amplitude. Latencies outside the a-priori 80-300ms poststimulus statistical window of interest are shown in parentheses to prompt interpretation with caution. n.s. = no significant points. Note: 496 is highest latency analysed.

	P50, Expt. 1	P50, Expt. 2	MMN, Expt. 1	MMN, Expt. 2	Late, Expt. 1	Late, Expt. 2
High Dev, High SD	*n*.s.	*n*.s.	86–156	121–164	(395–496)	(488–496)
High Dev, Low SD	n.s.	n.s.	(78)-195	98–215	(375–496)	(395–496)
Low Dev, High SD	(74–78)	n.s.	133–188	145–180	n.s.	n.s.
Low Dev, Low SD	(74)-82	n.s.	125–207	148–195	n.s.	(457–496)

### Differences in MMN between SD conditions

**Fig. 4:**
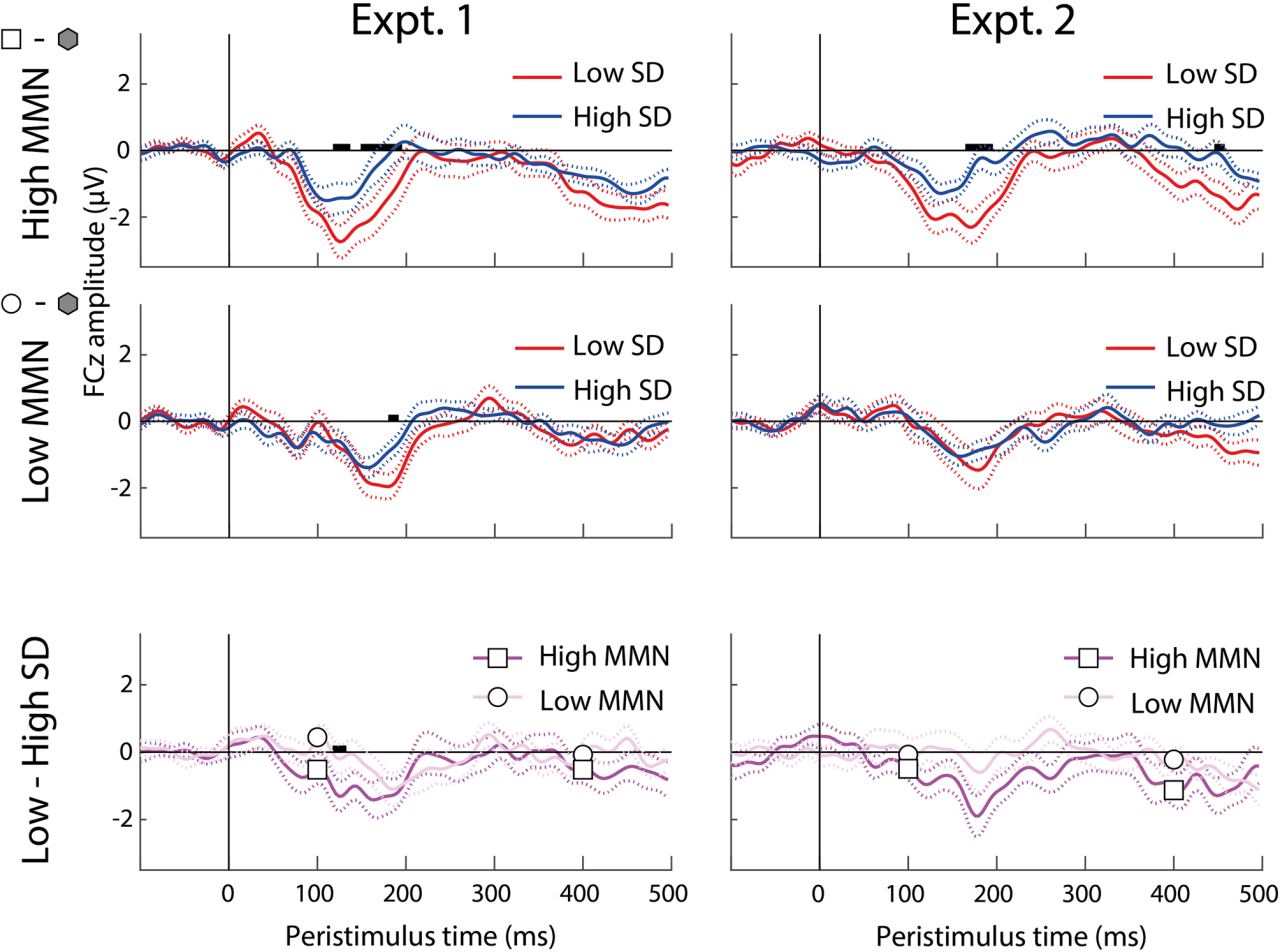
Comparison of mismatch negativity (MMN) responses between SD conditions. MMN waveforms were obtained by subtracting standard from deviant responses (as shown in Fig. 3). Dashed lines indicate standard error. ‘High MMN’ and ‘Low MMN’ refer to high and low deviants, respectively. ‘Low – High SD’ refers to the Low SD MMN minus the corresponding High SD MMN for a particular intensity of deviant. Black bars indicate time points of significant difference between the two MMNs shown in the plot, based on a non-parametric permutation analysis.

Figure 4 contrasts the MMN waveforms between equivalent SD conditions. In both experiments, for high intensity deviants, MMN was greater in the low SD than high SD condition at around 150-200ms. Exact latencies showing significant differences are summarised in Table 2. There was also a similar, but smaller and more temporally localised, difference for low intensity deviants in Experiment 1 only, whilst there were no differences between the two SD conditions for low intensity deviants in Experiment 2. However, these differences were not associated with a significant difference, in this timeframe, between the low SD minus high SD MMN modulation across the two intensities.

For high intensity deviants in Experiment 1 only, there was a larger MMN just after 100ms (coinciding with the second half of the N1 timeframe) in the low SD than high SD condition. This was associated with a significant difference between low SD minus high SD conditions between high and low intensity MMN.

For high intensity deviants in Experiment 2 only, low SD MMN was larger at around 450ms than high SD MMN.

**Table 2:** Latencies (ms) containing significant MMN differences between SD conditions. For High MMN or Low MMN, these were timepoints where Low SD MMN (i.e. deviant minus standard) amplitude was significantly more negative than high SD MMN for that deviant direction. For Low SD MMN – High SD MMN, timepoints are indicated where the amplitude of this contrast for high-intensity deviants was significantly more negative than for low-intensity deviants. Latencies outside the a-priori 80-300ms poststimulus statistical window of interest are shown in parentheses to prompt interpretation with caution. n.s. = no significant points. Note: 496 is highest latency analysed.

	P50, Expt. 1	P50, Expt. 2	MMN, Expt. 1	MMN, Expt. 2	Late, Expt. 1	Late, Expt. 2
High MMN	*n*.s.	*n*.s.	117–137, 148–195	164–195	*n*.s.	(445–457)
Low MMN	n.s.	n.s.	180–191	n.s.	n.s.	n.s.
Low SD MMN – High SD MMN	n.s.	n.s.	117–133	n.s.	n.s.	n.s.

## Discussion

**Fig. 5:**
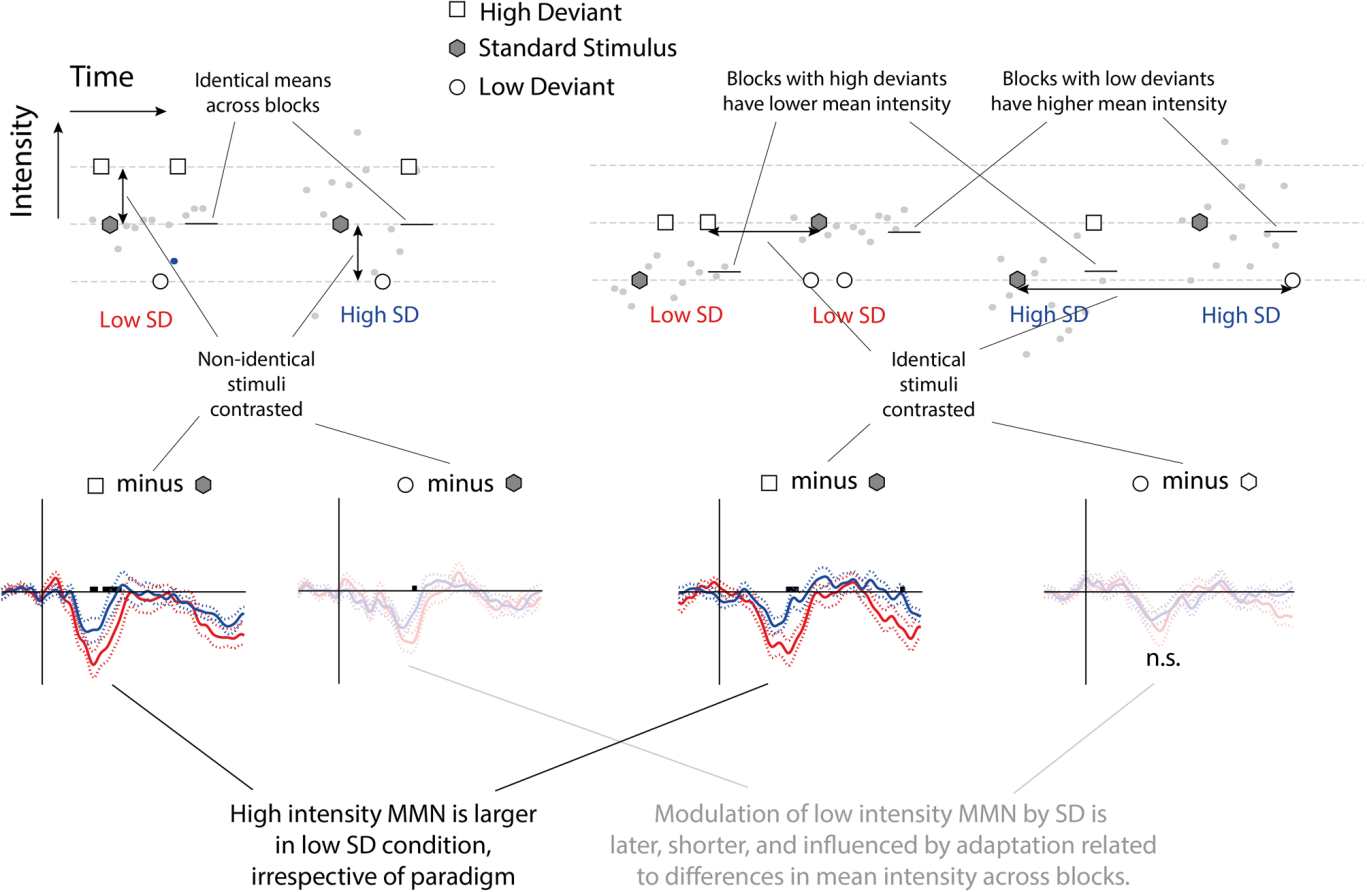
Summary of the two experimental paradigms and key significant results. Lower row of plots are mismatch negativity (MMN) responses following the same conventions as in Fig. 4.

A visual summary of the two experimental paradigms and key results is shown in Fig. 5.

### Summary and interpretation of findings

In this study, we sought to establish how intensity deviant MMN is affected by changes in contextual stimulus statistics. This was assessed in two similar experimental designs: one prioritising maintaining a more stable state of auditory adaptation (Experiment 1), and one prioritising contrasting deviants with physically identical standards (Experiment 2).

Firstly, we excluded differences in standard responses across statistical contexts as a potential confound. We then demonstrated robust MMN to both high (upward change) and low (downward change) intensity deviants in both experiments.

Smaller (less positive) P50 responses were seen for low intensity deviants (irrespective of SD condition) in Experiment 1 only, suggesting these can be explained simply as arising from weaker acoustic stimulation.

In the late N1 timeframe, only high intensity deviants elicited significant MMN, and this part of the MMN response was statistically modulated (i.e. larger in a low SD context), though this modulation was only significant in Experiment 1. Experiment 2 had a comparable non-significant trend, and a similar significant result might have been obtained with more participants. Our observations here are compatible with our other MMN studies of intensity that also find N1 timeframe responses only for intensity increases^14,15,19^, and concord with some other studies from the literature^11^. These findings may suggest that this early MMN component (overlapping with the end of the N1 timeframe) requires an increase in acoustic energy at stimulated frequencies to occur, but where it does occur it is modulated by stimulus statistical context. Alternatively, other processes than the generators of the N1 itself might affect MMN in this time window; for example, longer temporal integration dynamics in processing a reduction in intensity, or greater reliance on higher hierarchical levels of processing^14,15,19^.

In the classical MMN timeframe, both experiments yielded larger MMN for high intensity deviants in low SD compared to high SD stimulus contexts. This is consistent with previous findings for frequency changes^16^. Furthermore, the same sensitivity to statistical context was demonstrated in Experiment 1 for low-intensity deviants. This is an important observation, as it indicates that contextual modulation of MMN does not rely entirely on an increase in acoustic energy in particular frequency channels, and also cannot be fully explained by adaptive processes that only diminish neural firing, such as stimulus-specific adaptation, firing rate adaptation, repetition suppression or forward masking. The present results indicate overlapping mechanisms of statistical modulation of intensity deviants at these later, and possibly hierarchically higher, stages of processing, but that earlier stages are likely involved only for intensity increases.

Late negative deviant responses, around 300-400ms were seen in both experiments, Particularly Experiment 2, with some instances of modulation by stimulus statistical context, but as these were outside the time window of statistical interest, we simply suggest that this could be an area of subsequent study. Whilst less often reported, such components have sometimes been termed ‘late MMN’, for instance being reported when tone pairs, rather than single stimuli, deviate from an established pattern^20^.

Larger MMN to high intensity deviants in low-SD contexts was not simply an artefact of differing stimulus intensities between standard and deviant conditions, as it persisted in Experiment 2 where these two stimulus intensities were identical.

Interestingly, we observed differences in MMN to low intensity deviants between SD conditions in Experiment 1 only, and only a minimal non-significant trend in Experiment 2. This was initially somewhat surprising, as Experiment 2 involved subtracting physically identical standards, unlike the physically more intense standards in Experiment 1. We think it is unlikely that the increased direction-specific deviant frequency (i.e. same total number of deviants but all in the same direction) was responsible, as MMN to high intensity deviants in the classical MMN timeframe was highly similar across the two experiments, despite the same difference in deviant frequency.

Aside from the differences described above, the fact that high deviants and their corresponding standards were 6 dB quieter in Experiment 2 (but with no such differences for low deviants), and a mean stimulus intensity difference of around 0.1 dB (due to the asymmetry of the stimulus intensity distribution introduced by deviants of only one direction), the main differences between the two experiments relate to adaptation over longer timescales. Whilst adaptation of various sorts occurs over time ranges of milliseconds to tens of seconds, and therefore one would usually consider neural responses within the context of stimulus history over only these timescales (i.e. within one experimental block), there is also evidence for repetition-related plasticity occurring over timescales of 20 min or longer^21^, and regularity-related influenced on MMN amplitude ensuring over multiple minutes^22^. Once we move beyond within-block statistics, our two experiment paradigms become rather different. Experiment 1 maintains a constant stimulus mean over the entire experiment. Whilst its distribution changes, as the high and low deviants are close to the extremes of stimuli encountered, the approximate range of stimuli presented differs little across the whole experiment; longer-timescale adaptation should remain relatively constant. Conversely, in Experiment 2, the mean stimulus intensity varies between high and low mean intensity blocks, as does the range of stimuli encountered. For instance, in the low mean high SD block, stimuli below 50 dB are common, and some under 40 dB present. Furthermore, because the deviant intensity for one block type is the standard (or Gaussian mean) intensity from the other, any type of adaptation occurring and persisting over timescales of multiple blocks would be adapted to the deviants in Experiment 2 as highly familiar, making there little effective difference between the two SD conditions.

Based on our observations, and the arguments made in the previous paragraph, we suggest that there is a dissociation in mechanism between statistical context sensitivity to high (upward) and low (downward) intensity deviants, which may be driven by relatively more short-timescale and long-timescale predominant processes, respectively.

### Relevance to wider sensory function

Asymmetries in the processing of intensity changes are well-established^23^, and have been the result of considerable study. At both single-neuron level and perceptual/behavioural levels, upward intensity changes elicit larger responses than equal-sized downward changes. Evolutionary justification of this asymmetry is termed the ‘looming bias’^24^, which refers to the particular importance of detecting approaching entities in the environment, and has been shown to not be fully dependent on stimulus intensity.

We have recently shown, behaviourally, that the bias towards inferring increasing intensity of an ongoing stream of stimuli is particularly strong when that stream is more volatile (i.e. stimulus intensities have a higher SD), with this observation applying to both perceived salience of change and change direction discrimination performance^25^. One possible interpretation is that individual ‘outlier’ stimuli, particularly those that are unusually intense, are given disproportionately strong weighting in inferring mean intensity. Such an explanation could also apply to the results of the present study, both as an explanation for the modulation of MMN responses by stimulus statistical context at all, and also to explain the selective modulation of low intensity deviant responses only in experiments where such low intensity stimuli are uncommon overall; reactivity to intensity increases being a more fundamental and behaviourally imperative process may render it less susceptible to diminution by adaptation over longer timescales.

Increasing intensity of sounds is linked to greater perceived loudness and salience, which in turn lead to autonomic arousal, as commonly measured by pupil dilation response (PDR)^26^. During violation of auditory regularities, PDR has been found to correlate with MMN, independently of attention^27^, and, like MMN, is influenced by the preceding stimulus context^28^. To our knowledge, pupil responses to low (downward) intensity auditory deviants have not been specifically reported; however, in general, sound intensity decreases are associated with pupillary constriction rather than dilation. Whilst future work might specifically examine pupil responses to low-intensity deviants, for now we highlight the possibility that some of the asymmetries we have observed between intensity increases and decreases may relate to different effects on autonomic arousal and its associated neural processes. Future work using PDR in a paradigm similar to the present one (which has already been done for frequency deviants, showing a larger PDR in the low-SD context^29^, might establish whether the magnitude of high intensity deviant PDR is greater in a low SD stimulus context; if so, it could also indicate whether this is due to reaching a higher absolute peak level of arousal, or reaching a similar level from a lower arousal baseline.

Overall, our findings evidence a basis for asymmetry between the neural processing of intensity increases and decreases, where the latter are likely explained by processes operating over longer timescales and/or at higher hierarchical levels.

### Relevance to clinical disorders

Within the auditory domain, several common disorders centre around abnormal handling of sound intensity. Hyperacusis refers to the perception of sounds as uncomfortably or painfully loud that would not be to an average listener and can feature a particular sensitivity to sudden stimuli or intensity increases^30^. A distinct phenomenon is noise sensitivity^31^, whereby adverse consequences such as fatigue or concentration difficulties occur in environments with ongoing background sound. Phonophobia is the fear of loud sounds or noisy environments, and can co-occur with hyperacusis or noise sensitivity. Migraine commonly features one or more of these sound sensitivity symptoms, and similar sensitivities in other sensory modalities. Tinnitus is the perception of an ongoing sound in the absence of corresponding acoustic input. It can be thought of as the erroneous inference of a non-zero sound intensity at zero input intensity^32,33^. It is also commonly associated with hyperacusis. In the broader sense, across various deviant types, tinnitus is associated with reduced MMN amplitude^34^. However, intensity changes at tinnitus-related frequencies show a specific pattern, with hyperacusis (with or without coexisting tinnitus) associated with larger MMN amplitude to intensity increases, and isolated tinnitus with larger MMN amplitude to intensity decreases^19^. Thus, dichotomous findings between intensity increases and decreases can be of practical importance in disentangling the neural mechanisms of common clinical conditions. It would be of immediate interest to see whether intensity MMN differences in tinnitus and/or hyperacusis are dependent upon the stimulus context, by running this type of paradigm in these clinical groups; such findings might lend more specific detail about the kind of alteration in adaptation or attribution of sensory/prediction precision associated with these disorders, and differences between Experiment 1 and Experiment 2 versions might be additionally informative about the associated timescales of altered adaptive/predictive processing. Our present results reveal novel aspects of such intensity direction asymmetries, and add to the toolkit for studying disorders of sensory processing. The original frequency-varying version of the presently-used (Experiment 1) paradigm has already been found to produce significant correlations between the extent of statistical modulation of the MMN and self-rated sensory sensitivity^17^. Furthermore, chronic pain shares many clinical features and neural mechanisms with tinnitus^35–37^ and hyperacusis, and similar paradigms in the somatosensory domain may be used to study intensity processing in chronic pain conditions also.

### Limitations

We acknowledge several limitations. Firstly, the relatively small sample size and lower signal-to-noise ratio of EEG compared to MEG or intracranial EEG limited our power to resolve subtle effects, meaning there are some areas of uncertainty over whether particular effects were not present, or simply of small magnitude. Furthermore, this limited power led to an a-priori decision by us to limit the data space for analysis to focus on our hypotheses and specific research questions, rather than engage in more exploratory whole-scalp or whole-brain analyses to seek differences in the neuroanatomical basis of different deviant types. We constrained analyses to the time domain for the same reason. Additionally, our study was run only in healthy young participants, and findings may be different in older listeners, or those with hearing loss. We also did not control for, or manipulate attention, which again might influence findings.

## Conclusions

We have shown that MMN responses to acoustic intensity changes are modulated by the statistical context of preceding stimuli, as has been demonstrated for frequency and other auditory features. However, we add nuance to this area in showing that early MMN (overlapping with late N1) only occurs with an increase in acoustic energy, but is context-modulated when it occurs. MMN in the classical timeframe is robustly and consistently context-modulated for high intensity deviants, even when contrasted against physically identical standards. Conversely, context modulation of classical timeframe MMN to low intensity deviants is paradigm-dependent, and may be driven by adaptive processes over timescales of at least tens of minutes.

These observations further our understanding of the predictive and adaptive processes underpinning intensity and loudness perception, and may be useful in the study of numerous conditions characterised by aberrant processing of stimulus intensity, such as hyperacusis, migraine and chronic pain.

## Data Availability

Data are available on the Open Science Framework website via registration DOI https://doi.org/10.17605/OSF.IO/Y9MF3 which is linked to project https://osf.io/vn2dt.
